# Garlic Extract Increased Acrylamide Formation in French Fries Obtained by Different Cooking Methods

**DOI:** 10.3390/foods13172769

**Published:** 2024-08-30

**Authors:** Simge Sipahi, Timur Hakan Barak, Özge Can, Betül Zehra Temur, Murat Baş, Duygu Sağlam

**Affiliations:** 1 Department of Nutrition and Dietetics, Institute of Health Sciences, Acibadem Mehmet Ali Aydinlar University, Istanbul 34752, Türkiye; simge.sipahi@acibadem.edu.tr; 2 Department of Pharmacognosy, Faculty of Pharmacy, Acibadem Mehmet Ali Aydinlar University, Istanbul 34752, Türkiye; 3 Department of Biomedical Engineering, Faculty of Engineering and Natural Sciences, Acibadem Mehmet Ali Aydinlar University, Istanbul 34752, Türkiye; ozge.can@acibadem.edu.tr; 4 Department of Medical Biotechnology, Institute of Health Sciences, Acibadem Mehmet Ali Aydinlar University, Istanbul 34752, Türkiye; zehra.temur@live.acibadem.edu.tr; 5 Department of Nutrition and Dietetics, Faculty of Health Sciences, Acibadem Mehmet Ali Aydinlar University, Istanbul 34752, Türkiye; murat.bas@acibadem.edu.tr (M.B.); duygu.saglam@acibadem.edu.tr (D.S.)

**Keywords:** acrylamide, French fries, garlic, household preparation, air-fryer, oven-frying, pan-frying, HPLC

## Abstract

Fried potato products are the largest dietary source of acrylamide, a potential carcinogen formed at high temperatures. Previous studies suggested that garlic powder could decrease the development of acrylamide; however, there has not been much focus on the effect of garlic extract. The aim of this study was to investigate the effect of garlic extract exposure on the development of acrylamide in French fries in popular home cooking techniques such as pan-frying, air-frying, and oven-frying. Initially, the antioxidative profile, total phenolic content, and chlorogenic acid content of garlic were analyzed. Subsequently, potatoes were treated with garlic extract and fried using pan-frying, air-frying, and oven-frying techniques. Acrylamide levels were then quantified through HPLC and compared to control groups. The findings showed that garlic exposure increased the acrylamide formation in French fries obtained with air-frying (311.95 ± 0.5 μg/kg) and with oven-frying (270.32 ± 23.4 μg/kg) (*p* < 0.005 *). This study offers new insights into varying acrylamide formation levels in domestic practices. Unlike previous studies, this study is the first to question the effect of aqueous garlic extract exposure. Further research is required to comprehend the interaction between garlic exposure and acrylamide formation in household settings.

## 1. Introduction

The discovery of acrylamide (AA) dates back to 2002, as an end-product of Maillard reactions occurring between free amino acid asparagine and reducing sugars, formed in starchy foods such as potatoes and cereal grains when prepared at temperatures above 120 °C and low moisture. These substances have been shown to be genotoxic and carcinogenic to rats; thus, they are classified as 2A carcinogenic effects in humans, according to a statement released by the International Institute for Research on Cancer (IARC) [1]. Even though additional pathways have been proposed to contribute to AA production, the main formation route of AA in foods is accepted to be through Maillard reaction, and fried potato products are the greatest source of AA of the diet, according to the European Food Safety Authority (EFSA) [2].

Potato is the fourth most important staple food for humans as a versatile ingredient with different heat-processing techniques to choose from such as baking, boiling, or frying [3]. Despite the acrylamide content in French fries, the consumption of the product has increases, and they are widely used around the world by consumers, restaurants, and industries [4]. The disequilibrium between the possible harms and popularity of French fries leads researchers to work on improving fried products in terms of reducing oil content and newly generated harmful products during food processing, mainly by applying alternative frying methods. In our study, three different cooking methods that are frequently used in households were preferred: pan-frying, air-frying, and oven-frying. The air fryer uses hot air for heat transfer, creating a healthier alternative with higher flavor and using approximately 90% less oil than the alternative pan-frying, and it is thus considered the first alternative to pan-frying with growing popularity. The second alternative is the oven-frying method, in which hot air convection is used with some secondary radiation coming from the oven walls and some conduction coming from the baking tray. Even though both methods are commonly preferred in both household and industrialized setups, there remains a lack of understanding about acrylamide formation in products prepared with them [5,6].

Approximately half of the total acrylamide intake is related to household practices, catering services, and restaurants. Within those routes, the existing large variance and high acrylamide contents in French fries appear to be mostly caused by the lack of standardized frying control and the various food handler behaviors. The acrylamide content obtained from household practices varies greatly and is approximately double the average value that EFSA took into account [7]. The main causes of the wide range and high acrylamide contents in French fries in both household and catering settings appear to be the unstandardized food-handling procedures and the absence of regulated control over the frying temperature and time (resulting from inadequate frying equipment) [8]. Nonetheless, when consumers are informed about the acrylamide content of fries, they tend to choose lower acrylamide-containing options [9]. Therefore, it is two-sided work: analyzing the household consumption levels and trying to come up with risk-reducing strategies

A variety of techniques are studied to lower the levels of acrylamide in food, and one of the most popular and promising ones is the use of inhibitors. It is believed that the presence of antioxidants, particularly those classified as inhibitors, may react with the intermediate products of the Maillard reaction to limit the amount of acrylamide. Thus, it is believed that using antioxidants naturally found in plants could be a useful tactic for lowering the production of acrylamide in food [10,11]. However, there is a gap in the literature regarding the exact mechanism underlying the possible impact of antioxidants, one of which is garlic. Garlic (*Allium sativum* L.) is a widely used culinary component in many recipes and a significant crop for economies of various nations [12]. It has long been vital to human nutrition and has been linked to a lower risk of developing a number of diseases, according to epidemiological, clinical, and preclinical research [13]. Apart from these effects, raw garlic has strong antioxidant qualities as well [14]. Previous studies demonstrated that garlic could have an inhibitory effect on acrylamide formation, mainly attributed to its allicin content; however, these studies employed the plant as a powder and were usually conducted on models, not in household settings [15,16]. Yang et al. found that garlic is also effective in the inhibition of heterocyclic amines, other carcinogenic and/or mutagenic Maillard reaction products formed in sausages [17]. Despite extensive research on the effect of garlic powder on the development of acrylamide, little attention has been paid on garlic extracts, a route more applicable to domestic conditions. Aqueous garlic extract was chosen regarding its easier reproducibility and acceptability in household settings. Therefore, in this study, garlic extract was used in French fries obtained from different cooking practices common among household settings to determine its impact on the formation of AA.

The aim of this study was to investigate the effect of garlic extract exposure on AA formation in different cooking practices that are common among households. In the first part of the study, the antioxidative profile, total phenolic content, and, specifically, chlorogenic acid content were analyzed. In the second part, potatoes were treated with the aqueous extract prepared with the analyzed garlic samples prior to being fried in different cooking techniques, preparing an environment where the formation of acrylamide can be observed. For the latter part, the formed acrylamide levels were quantified and compared in terms of differing the effect of garlic exposure contrasted with the control groups.

## 2. Materials and Methods

### 2.1. Chemicals and Materials

The potatoes and sunflower seed oil were purchased from the local supermarket (Metro Grossmarket, Istanbul, Türkiye). When purchasing potatoes, care was taken to ensure that they were harvested in the same season, sold by the same seller, with identical growing conditions, and transported under appropriate conditions. The cultivar/variety is an important factor affecting the amount of reducing sugars and asparagine of potatoes and, consequently, acrylamide formation in products; therefore, potatoes were selected from the same cultivar (*Agria*), not for the cultivar specifically but to exclude any confounding factor that a new cultivar may bring [18]. Until the analysis, purchased potatoes were stored in boxes in a cold storage room (10 °C). The same type of oil was also preferred in order to surpass the possible differences in acrylamide formation [19]. The garlic samples, grown with identical conditions and harvested in the same month, were collected from a grower in Taskopru, Türkiye (ACT Sarimsak, Kastamonu, Türkiye). All solvents and chemicals were purchased from Sigma-Aldrich (Steinhein, Germany).

### 2.2. Preparation of Garlic Extract

**Figure 1:**
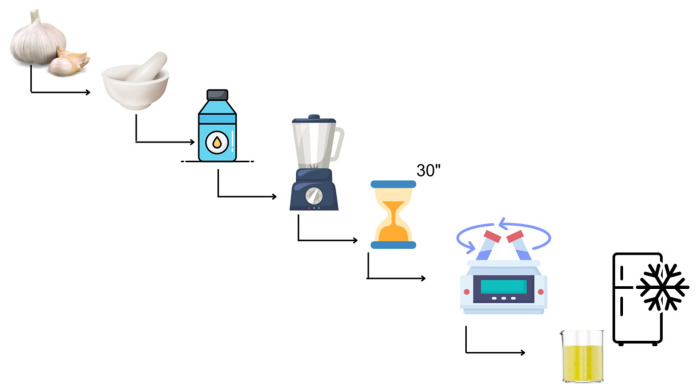
Preparation and storage flow chart of aqueous garlic extract.

Garlic extracts were prepared using distilled water as the solvent. Distilled water performed better than ethyl alcohol and acetone for reflecting the total antioxidant capacity [20]. In this sense, the technique employed by Sermenli was used to obtain the garlic extracts [21]. The garlic (625 g) was crushed and homogenized in distilled water (1:1) equal to its own weight with the help of a blender. Centrifugation was applied for five minutes at 6000 rpm after waiting for thirty minutes at room temperature. After the mixture was centrifuged, the supernatant was collected and kept at 4 °C for further analysis. The preparation step of the extract is summarized in Figure 1.

### 2.3. Determination of Total Phenolic Content (TPC)

The method of Bardakci was followed in evaluating the total phenolic content of the garlic extract [22]. The mixture of 20% Na_2_CO_3_ and FCR (Folin-Ciocalteu reagent diluted with H_2_O (1:9)) was added to the diluted extract sample. Following a 30 min incubation period at 45 °C, the absorbance of the solution was determined at 765 nm. Gallic acid equivalents (GAE) per gram of dry extract (DE) were used to express the results. Spectrophotometric calculations were conducted using a Thermo Multiskan Sky Microplate Spectrophotometer (Waltham, MA, USA).

### 2.4. Quantification of Chlorogenic Acid by HPTLC

The quantitative and qualitative measurement of chlorogenic acid was performed by an HPTLC system according to the previously used method [23]. For this, 20 cm × 10 cm HPTLC silica gel 60 F 254 plates were used. The mobile phase was determined as the mixture of EtOAc-CHCI_3_-FA-AA-H_2_O with a volume of 100:25:10:10:11. The samples were dissolved in MeOH at a 1 mg/mL concentration, while standards were also prepared in MeOH at a concentration of 100 µg/mL. Using the CAMAG Automatic TLC Sampler IV, extracts were applied at increasing amounts (2–40 μL) in 6 mm long bands with a minimum of six concentrations of the standard solution (2–12 μL). Developments were made in the CAMAG Automatic Development Chamber-2 (ADC-2). The chamber was saturated for 10 min and humidity was controlled by ADC-2 with MgCl_2_ (33% relative humidity). After derivatization with Natural Product Reagent (NPR), densitometric evaluation was performed using a CAMAG TLC Scanner IV in fluorescence mode. The amounts of the standards in the sample were obtained by a comparison of AUCs with the calibration curve of the standards. The visionCATS software, version 2.0 (Camag, Muttenz, Switzerland) was used for image processing. The correlation coefficients (r^2^) were found to be >0.998 for the analysis of the samples.

### 2.5. Evaluation of Antioxidant Activity of Garlic Extract

To analyze the antioxidative capacity of garlic samples, a DPPH radical-scavenging activity test was performed, in accordance with a previously published method [24]. After obtaining a combination of freshly diluted sample solutions with different concentrations prepared from 1 mg/mL stock solution and methanolic DPPH solution (100 mM), the samples were incubated for 45 min at room temperature. The absorbance was read at 517 nm. For the reference compound, butylated hydroxy toluene (BHT) was used in acquiring a calibration curve. The results were expressed as the mean of the triplicates ± standard deviation (S.D.) and as mg butylated hydroxy toluene equivalents (BHTE) in 100 mg total weight.

### 2.6. Preparation and Quantification of Acrylamide

#### 2.6.1. Frying Experiments

The potatoes were washed, peeled, and manually cut into 7 × 7 mm strips. This size of potato strips has been reported to have the highest acrylamide content [25]. After cutting, they were submerged in running water for a minute to remove any remaining starch. The potatoes were then patted dry with a paper towel.

**Figure 2:**
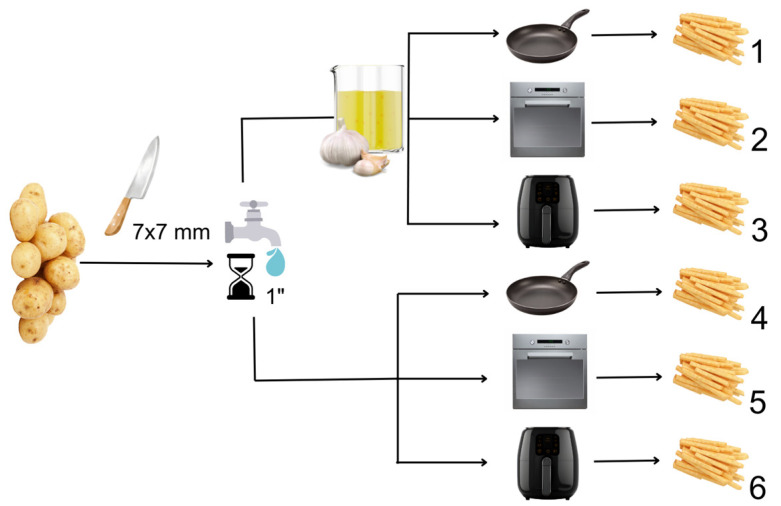
The preliminary and frying steps of potatoes for each sample groups: (1) pan-fried French fries with aqueous garlic exposure; (2) oven-fried French fries with aqueous garlic exposure; (3) air-fried French fries with aqueous garlic exposure; (4) control group for pan-fried French fries; (5) control group for oven-fried French fries; (6) control group for air-fried French fries.

Following the preliminary step, the potatoes were divided into two separate groups, one group was kept in garlic extract for a minute, whereas the other group was kept as a control group without any intervention. Both groups were separated into three individual subgroups for cooking using pans, an air fryer, and an oven. The pan-frying method was selected since it is a suitable approach for household cooking practices. The amount of oil that covered the potatoes was added (with 1:1 ratio per weight). The temperature of the oil was monitored with a food thermometer (Thermopro TP01H food thermomether (ThermoPro, Shenzhen, China), and the target temperature was determined as 180 °C. When the temperature was measured at 120 °C, the potatoes were immersed in the oil and were removed from the pan at the fifth minute. The temperature of the potatoes and the oil was measured one minute apart [25]. In an air fryer, the potatoes were placed in such a manner as to not come into contact with each other. To guarantee that the target temperature of 180 °C was reached, the internal temperature was continuously checked using PT100-type temperature sensor probes located both at the top and bottom of the chamber. The temperature measured by the probes was read by the outside connected screen. The use of a continuous thermometer increases the quality of the work by ensuring that the temperature used as the main driving force is kept at the desired level throughout the process. Oil was added to the potatoes in a proportion equivalent to one tablespoon of oil per kilogram. After setting the air fryer to 180 °C and achieving the target temperature, the potatoes were added, and they were taken out after 21 min [5]. In the oven frying method, 12 g of oil was added per 1 kg of potato, using a spray for homogeneous distribution. The oven temperature was set to 180 °C and controlled continuously with the same thermometer sensor probes, and the potatoes were removed from the oven after 30 min [6]. Absorbent paper was used in all groups to drain excess oil on potatoes after frying. To obtain samples with comparable sensory qualities in terms of color and texture, different frying times in different cooking techniques were chosen, each determined by previous studies. However, to overcome any possible further differences between different frying equipment, the frying temperature was set at 180 °C. The frying procedure is summarized in Figure 2.

#### 2.6.2. Extraction of Acrylamide

A modified extraction procedure that was adapted from the method developed by Khoshnam et al., 2010 was used [26]. Briefly, 4 g of finely ground, homogenized French fried potatoes was weighed in a closed flask, which was then defatted twice by adding 10 mL hexane and shaking for five minutes. Following decantation, the mixture was dried under vacuum. The defatted sample was mixed with 100 μL of distilled water, and 20 mL of acetone was added to extract AA. The flask was submerged in an ultrasonic bath set at 40 °C for 20 min. A filter paper was used to filter the acetone. A total of 20 mL of the filtrate was carefully dried by vacuum evaporation. To dissolve the residue, 2 cc of distilled water was added and vigorously shaken. After passing through a filter paper, the aqueous solution was injected into glass vials. The samples were prepared in triplicate.

#### 2.6.3. HPLC Analysis of Acrylamide

The quantification of acrylamide was employed with the method of Haddarah et al., with some modifications [27]. The high-performance liquid chromatography used for detection was the 1260 Infinity model of a liquid chromatography system, 110 quaternary pumps (DEAB804078), 1260 thermostat column compartment (DEACN19021), Agilent injector with a loop with a 40 μL volume, 1260 diode array, and multiple wavelength detector (DAD) (DEAAX02373). The mobile phase solvent was acetonitrile with a ratio of acetonitrile to pure water of 5/95 and a flow rate of 0.5 mL/min. The method comprised an ACE 5 RP-C18 analytical column (25 cm × 4.6 mm, 5 μm particle size), a flow rate of 0.5 mL/min, detection at the 202 nm wavelength, and an injection volume of 40 μL. A mean standard curve was used to estimate the level of acrylamide content (20, 80, 160, 320, and 400 ng/mL). Each solution was analyzed in triplicate, and the mean values were used for calibration.

### 2.7. Statistical Analysis

The statistical analyses were carried out using Graphpad Prism software version 8. The mean values of triplicates (*n* = 3) from the three different runs make up all the data displayed. The statistical significance was established at *p* ≤ 0.05. The Tukey’s multiple comparisons test was used to identify significant differences among different groups.

## 3. Results

### 3.1. Total Phenolic Content of Garlic Extracts

The total phenolic content of garlic extracts is given in Table 1. These results are higher than the findings of Jang (5.68 ± 0.51 mg GAE/g) and Koca (1.59 mg GAE/g), whereas they are lower than the findings of Nuutila (75–700 mg GAE/kg) and Gorinstein (19.40 ± 1.2 mg GAE/g) [28,29,30,31]. These differences may be attributable to the genotype, cultivation practices, harvest time, and climatic conditions [32]. The use of pure water was effective in obtaining aqueous garlic extracts with high total phenolic compounds, which aligns with the results of this study as well [20]. These results demonstrate the rich total phenolic content of garlic extract, which may also exacerbate antioxidative activity.

### 3.2. Antioxidant Scavenging Activity

In our study, the antioxidant activity was evaluated by DPPH radical scavenging activity, a method based on the principle of DPPH radical disappearing with the acceptance of an electron from an antioxidant molecule. Garlic samples exhibited moderate DPPH radical scavenging activity, measured as 765 ± 4 mg BHTE (Table 1). The antioxidative profile of garlic extract was a key factor to demonstrate prior to the intervention since garlic was chosen as an antioxidant ingredient that may interfere with the acrylamide formation pathway. Akan compared French, Spanish, Chinese, and Turkish Taskopru garlics, where Taskopru garlic showed the highest antioxidant activity (62.58%) [32]. Taskopru garlic, also known as “white gold”, is the most popular garlic clone in Türkiye [33]. The garlic samples used in the study were purchased from the grower four months after their harvest. The storage time influenced the antioxidant capacities in garlic cloves, showing a maximum level in the 8th week and continuing to decrease afterwards in a study conducted by Li et al., which may explain the lower results obtained in this study [34]. The manufacturing process, the temperature and drying time, and the use of polar or nonpolar extraction solvents all have a significant impact on the chemicals found in garlic products [35].

### 3.3. HPTLC Analysis of Chlorogenic Acid Content

**Figure 3:**
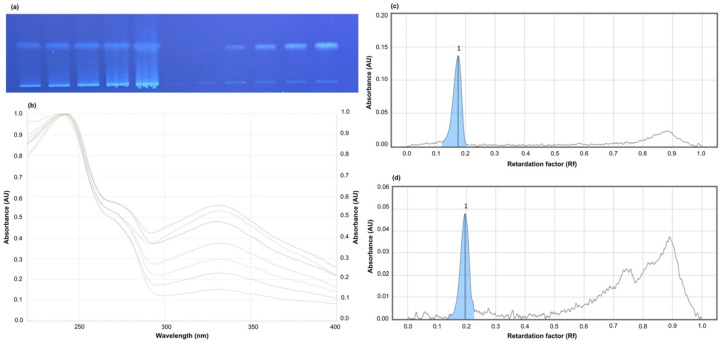
HPTLC Analysis of the extracts. (**a**) Visualization of the plate (Garlic extract and chlorogenic acid standard); (**b**) UV spectra of chlorogenic acid and same Rf value of tracks at 200–450 nm; (**c**) HPTLC Chromatogram of the standard; (**d**) HPTLC Chromatogram of the extract. Mobile phase: EtOAc-CHCI_3_-FA-AA-H_2_O (100:25:10:10:11) Derivatization: NPR reagent. Visualization: 366 nm.

The chlorogenic acid level of the garlic sample was expressed as µg/g dry extract in Table 2 and Figure 3. A wide range of polyphenolic compounds are present in garlic. Snirc determined chlorogenic acid as 17.49–20.2 mg/kg, whereas Yünlü reported the chlorogenic acid content of his garlic samples as 1.6 μg/g [36,37]. Furthermore, the phenolic composition of crops may vary according to their genotype, agronomic conditions, environmental factors, maturity, and post-harvest processes, a possible explanation for the variations in results among studies [38]. The quantification of chlorogenic acid in garlic extracts was important in better interpreting the results of acrylamide analysis with the profile of garlic.

### 3.4. Acrylamide Content

#### 3.4.1. Effect of Fryer Type on Acrylamide Formation

**Figure 4:**
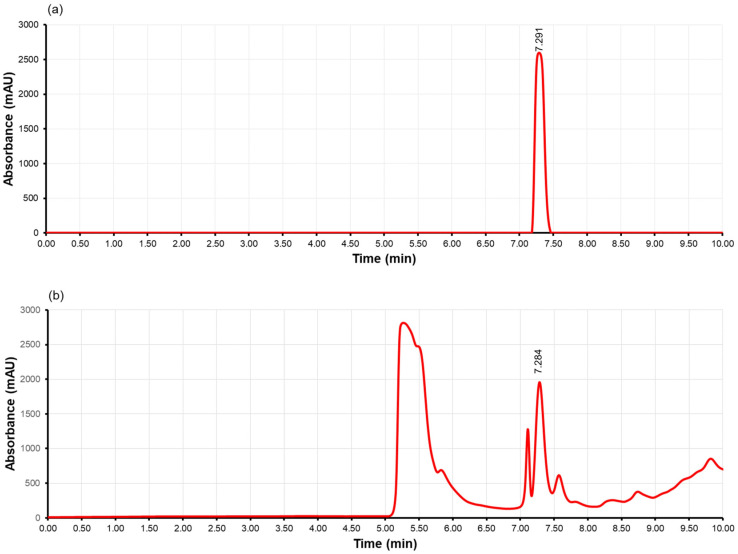
HPLC chromatograms of (**a**) acrylamide standard and (**b**) test sample at 202 nm.

**Figure 5:**
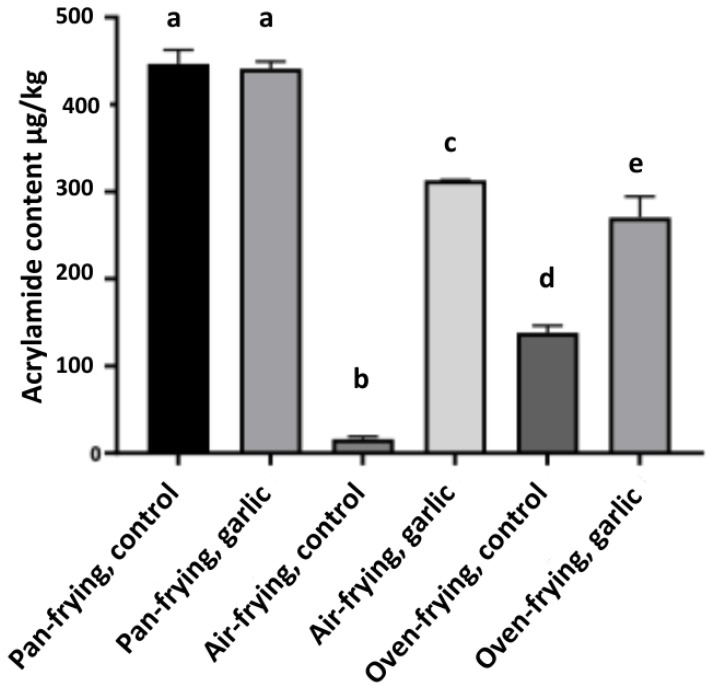
Acrylamide content of French fries according to cooking techniques and garlic extract exposure. Different letters indicate significance (*p* < 0.05).

The occurrence of acrylamide in the test samples was verified with the presence of a concurrent peak matching the retention time observed in the acrylamide standard. An example chromatogram demonstrating both the chromatogram of a sample and the chromatogram of the acrylamide standard is presented in Figure 4. Both peaks of the acrylamide standard and test sample are detected at a 7.29 min retention time. The calibration curve of the AA standard was linear (R^2^ = 0.9968). The verified acrylamide quantities of the samples are presented in Table 3 and Figure 5. An objective of this study was to investigate the impact of thermal processing methods on acrylamide formation. There was a significant difference in the formation of AA for the control pan-fried French fries (446.76 ± 15.87 μg/kg) as opposed to the air-fried or oven-fried ones (16.06 ± 3.05 μg/kg, and 138.34 ± 8.07 μg/kg respectively). The amount of AA formed varied significantly between the three types of frying (*p* < 0.01). These results may be useful when choosing a lower acrylamide-forming alternative in domestic conditions. The current literature suggests that air-frying produces lower amounts of acrylamide compared to deep-frying, consistent with the findings of the present study [39]. Dong et al. found that air-frying reduced acrylamide and 5-hydroxymethylfurfural contents, with a maximum reduction rate of 57.04% and 47.31%, respectively, compared to deep-frying [40]. In another study conducted by Sansano et al., air-frying reduced the acrylamide content in fried potatoes by about 90% in comparison to that of conventional deep-fried potatoes [41]. In contrast to the aforementioned results, Navruz-Varlı and Mortaş observed an increased acrylamide formation in potatoes fried in an air fryer (12.19 ± 7.03 μg/kg) compared to deep-frying (8.94 ± 9.21 μg/kg) and oven frying (7.43 ± 3.75 μg/kg); however, the difference among groups was not statistically significant [42]. A possible explanation for these contradictory results can be made through the choice of different frying temperatures among different equipment and inadequate frying times (especially in oven-cooking technique, resulting in an uncommon frying end-product). In planning a research design that is appropriate for domestic behaviors, the decisions of consumers are particularly crucial.

**Figure 6:**
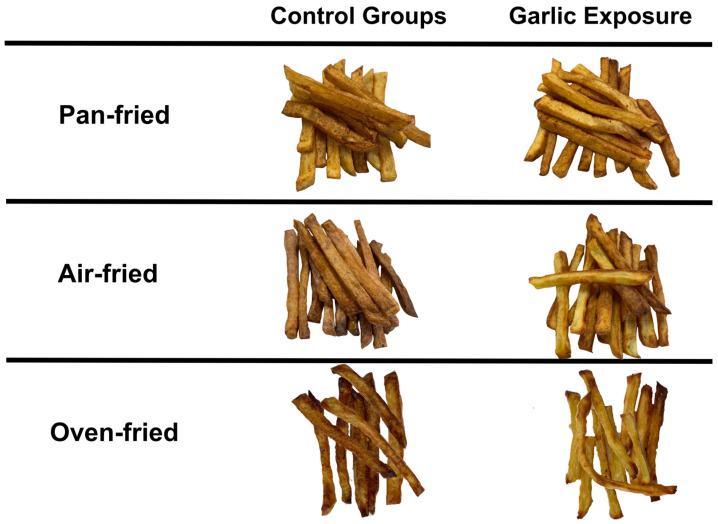
Visual representation of French fries with or without garlic exposure according to different cooking methods.

The variation in acrylamide formation between different cooking techniques is attributed to the differences in oil quantities used in these cooking techniques. Acrylamide development increases in fried foods containing more than 50% oil by weight [43]. With its mechanism, the oil uptake is much lower in air frying in comparison to deep-frying and oven-frying [5]. Apart from air-frying, oven-fried potatoes generated lower quantities of acrylamide compared to pan-fried potatoes, aligning with previous findings as well [44]. To visually illustrate the effect of garlic extract on color changes of French fries, images taken from both the control and sample groups are presented in Figure 6. The degree of Maillard reaction is usually reflected in the color changes of food surfaces [40]. Nevertheless, in our study, no observational relationship could be made between the color of French fries and the amount of acrylamide. For further and more comprehensive understanding, a colorimetric analysis better be involved in future study designs.

#### 3.4.2. Effect of Garlic Extract on Acrylamide Formation

Numerous plant extracts showing antioxidant profiles are reported to cause a significant decrease of up to 60% in acrylamide formation through possibly interacting with the precursors of acrylamide in the acrolein and asparagine pathways [45]. Natural polyphenols, as an alternative to synthetic antioxidants, are also preferred to decrease the acrylamide formation due to their radical scavenging activity, carbonyl trapping effect, and restriction of sugar degradation via the Maillard reaction [46]. When added as an exogenous additive, polyphenols were also able to decrease the formation of acrylamide in baking products [47]. Nevertheless, recent advancements in the field have revealed contradictory evidence on the effect of antioxidants and polyphenols on the formation of acrylamide. A study aiming to observe the capacity of a widely used antioxidant, curcumin, during acrylamide formation found that curcumin contributed to acrylamide formation; this effect was linked with the ability of the carbonyl group present in antioxidants and its reactivity with asparagine leading to the formation of acrylamide [48]. There are contradictory results showing the acrylamide formation enhancer effect of polyphenols as well [49,50]. Likewise, conflicting results associated with antioxidants, polyphenols, and acrylamide formation were also observed in our study. When compared with the control group, garlic exposure increased the acrylamide formation in French fries obtained with air-frying (16.06 ± 3.05 μg/kg and 311.95 ± 0.5 μg/kg, respectively), and this was also true compared with those obtained with an oven (138.34 ± 8.07 μg/kg and 270.32 ± 23.4 μg/kg, respectively) (*p* < 0.005 *). On the other hand, in pan-frying, no statistical significance was found between the two groups (446.76 ± 15.87 μg/kg and 441.21 ± 8.08 μg/kg, respectively).

One of the most important attributors behind our results may be the effect of chlorogenic acid. The addition of the previously quantified chlorogenic acid through garlic exposure is observed to significantly increase acrylamide formation. The chlorogenic acid content present in the garlic extract may have cause an upward trend in the acrylamide content of French fries exposed to garlic extract. Correspondingly, Cai et al., reported that the addition of chlorogenic acid in the asparagine/glucose Maillard reaction system resulted in significantly increased acrylamide formation. This effect was associated with the ability of chlorogenic acid to increase the formation of a more efficient precursor (hydroxymethylfurfural (HMF)) in the formation of acrylamide, to decrease the activation energy for the conversion of 3-aminopropionamide (3-APA) to acrylamide, to enhance deamination from 3-APA, and to prevent the attack of the acrylamide generated by free radicals. When co-heated with asparagine, HMF is able to form more acrylamide than glucose; hence, with increased levels of newly formed HMFs, an increased formation of acrylamide may also be observed. On the other hand, 3-APA, another precursor transforming to acrylamide following the deamination process, was observed to require a lower activation energy and enhanced deamination process with the presence of chlorogenic acid, another adjustment resulting in the increased formation of acrylamide. Free radicals produced by the Maillard reaction have the ability to destruct acrylamide; however, chlorogenic acid raised the redox potential of the system and stopped acrylamide from being eliminated through oxidation and free radical reactions [51].

Garlic is shown to have varying levels of phenolic compounds. Both thiosulfinates and phenolic compounds are recognized for their good antioxidant activity in garlic [18,52]. Many studies observing various activities of garlic were built around the main active substances in garlic, thiosulfinates and sulfur-containing compounds, produced by the action of alliinase from alliin. With the enzymatic reactions catalyzed by alliinase, allicin is formed from alliin after the activation of the enzyme by the crashing of the bulb of garlic [12,15]. The first study focusing on the main active substance of garlic powder, allicin, found that allicin was observed to be effective in reducing the formation of acrylamide when used in the form of garlic powder. An even higher acrylamide reduction rate was observed when the garlic powder was obtained through freeze-drying compared to oven-drying. This diminishing effect of garlic powder was attributed to the antioxidant property of allicin, which inhibits the reaction of amino acid and asparagine and results in decreased Schiff base formation [15]. In another study, garlic powder effectively lowered AA formation when added in an asparagine/glucose low-moisture model system as well, with an AA reduction rate of 43% [16]. In the present study, instead of garlic powder, garlic extract was used for its applicability in household settings. The choice of garlic extract over garlic powder might be the cause of this contradictory result. Garlic extract still has allicin as one of the major compounds; nevertheless, the allicin level may vary between different garlic products such as extracts or powder [53]. This research was the first study to use aqueous garlic extract in different cooking techniques for analyzing the acrylamide formation level in French fries.

While this study provides insights into the impact of garlic treatment on acrylamide formation, its effect on the bioavailability of acrylamide was neglected. Following oral intake, unbound AA is absorbed from the gastrointestinal tract and rapidly distributed systemically into the tissues circulation, where it is metabolized to form glycidamide (GA), the major accepted route for its genotoxic and carcinogenic profile [2]; thus, it is of great importance to decrease the bioavailability of acrylamide. Martinez et al. observed the bioaccessibility of acrylamide and found that, specifically during the gastric phase of the digestion, newly generated small peptide chains and some amino acid residues such as cysteine and lysine, all related to the action of pepsin, have the ability to interact with acrylamide and form adducts, resulting in a decreased level of acrylamide absorption [54]. In another study, Mechi et al. used olive leaf extracts to compare the acrylamide in Californian-style black olives. There was no difference in gastrointestinal digestion, but the acrylamide quantity decreased when compared with that of the control group, an effect that was related to the increased antioxidant activity [55]. When it comes to acrylamide originating from French fries, the outcome changes a little; Hamzalıoğlu and Gökmen found that acrylamide from French fries showed a noteworthy increase after gastric digestion. This increase was associated with the possible conversion of intermediates like the Schiff base accumulated in potatoes to acrylamide during gastric digestion [56]. Therefore, the effect of garlic treatment on acrylamide absorption is an important area for investigation before concluding its ultimate outcome in terms of acrylamide exposure.

## 4. Conclusions

The effects of garlic extract on acrylamide levels in French fries were examined. It was shown that aqueous garlic extract demonstrated the potential to increase acrylamide formation in French fries (*p* < 0.001), in contrast to previous findings about the effect of garlic powder. This study will enrich the conflicting literature by being interpreted as an example for the disadvantageous effect of antioxidant ingredients in acrylamide formation, especially through the effect of chlorogenic acid. The current study presents the first results regarding the use of aqueous garlic extract in different cooking techniques for analyzing the acrylamide formation level in French fries. For a complete evaluation of their effect, garlic extracts with different concentrations are required.

This study sheds light on the variations in acrylamide formation in different up-to-date cooking techniques in a matching manner with the domestic conditions, an underexplored area in the literature. Based on the findings focusing on the choice of frying methods, the amount of AA formed varied significantly between the three types of frying (*p* < 0.01). In agreement with previous studies, air-frying was observed to produce the lowest acrylamide levels. Using an air fryer may be a useful strategy for opting to a lower acrylamide-forming alternative. However, more informative recipes involving various frying utensils can be prepared to reduce the acrylamide intake related to household practices.

The major limitation of this research is that only the main route of acrylamide formation, the Maillard reaction, was evaluated. This approach can be criticized for not observing the other route for acrylamide formation thoroughly, from asparagine involving 3-APA, a route that enables acrylamide production without reducing sugars/catalysts. Further studies are needed to conclude the effect of different additives in acrylamide formation in French fries through different cooking techniques.

## Figures and Tables

**Table 1:** Total phenolic content and DPPH radical scavenging activity of garlic.

Sample Name	Total Phenolic Content *	DPPH ** Radical Scavenging Activity
Garlic	15.0 ± 0.5	765.0 ± 4.0

* Results were expressed as the mean of triplicates ± standard deviation (S.D.) and as mg gallic acid equivalents (GE) in 100 mg total weight. ** Results were expressed as the mean of the triplicates ± standard deviation (S.D.) and as mg butylated hydroxy toluene equivalents (BHTE) in 100 mg total weight.

**Table 2:** Quantification of chlorogenic acid with HPTLC.

Compound	Amount	CV (%)	R^2^
Chlorogenic Acid *	5.74 ± 0.06	0.98%	0.99

* Result was calculated as µg/g dry extract. CV: Coefficient of variation.

**Table 3:** Acrylamide content of French fries in different cooking methods.

Cooking Method	Garlic Treatment	Acrylamide Content (μg/kg)		
		Mean ± SD	*p*-Values **	*p*-Values ***
Pan-frying	Control	446.76 ± 15.87 ^a^	*p* = 0.99	a–c, *p* < 0.0001
	Garlic	441.21 ± 8.08 ^b^		a–e, *p* < 0.0001
Air-frying	Control	16.06 ± 3.05 ^c^	*p* < 0.0001 *	c–e, *p* < 0.0001
	Garlic	311.95 ± 0.49 ^d^		b–d, *p* < 0.0001
Oven-frying	Control	138.34 ± 8.07 ^e^	*p* < 0.0001 *	b–f, *p* < 0.0001
	Garlic	270.32 ± 23.38 ^f^		d–f, *p* = 0.01

The superscript letters “a, b, c, d, e, f” cite whether there is statistical significance for similar treatments in different cooking methods. * *p* < 0.001. ** The *p*-values express the difference between control and garlic groups in the same cooking method. *** The *p*-values express the difference among different cooking methods in the same control or garlic exposure.

## Data Availability

The original contributions presented in the study are included in the article, further inquiries can be directed to the corresponding author.
